# A pre-Inca pot from underwater ruins discovered in an Andean lake provides a sedimentary record of marked hydrological change

**DOI:** 10.1038/s41598-019-55422-1

**Published:** 2019-12-16

**Authors:** Neal Michelutti, Preston Sowell, Pedro M. Tapia, Christopher Grooms, Martin Polo, Alexandra Gambetta, Carlos Ausejo, John P. Smol

**Affiliations:** 1 Paleoecological Environmental Assessment and Research Laboratory (PEARL), Department of Biology, Queen’s University, Kingston, Ontario, Canada; 2 Geotic Solutions, Boulder, Colorado, USA; 3 INAIGEM – Dirección de Investigación en Ecosistemas de Montañas, Huaraz, Perú; 4 El Centro Peruano de Arqueología Marítima y Subaquática [CPAMS], Lima, Peru; 5 Kullasiri S.A.C., Lima, Peru

**Keywords:** Limnology, Palaeoecology

## Abstract

Pre-Hispanic artifacts and sacred architecture were recently discovered submerged in a large lake (Laguna Sibinacocha) in the Peruvian Andes. The underwater ruins indicate a dramatic shift in the region’s hydrology but the timing and triggers of this shift remain unknown. In a novel approach blending archaeology and paleoecology, we analyzed a sediment sequence from within one of the recovered artifacts, specifically a pot from the Late Intermediate Period (~1000–1400 CE). Radioisotopic dating of discrete sediment intervals sampled from the pot show a stratigraphically intact profile that preserves a history of change at this site. The pot’s basal sediment age places the timing of lake-level rise at ~1600 CE, which post-dates the end of the Inca Empire (1400–1532 CE) by several decades. The ubiquity of planktonic algae throughout the sediment profile suggests water levels remained high above the pot since its submergence. Paleoclimate data from the nearby Quelccaya ice core records indicate lake flooding followed a pronounced wet period beginning ~1520 CE. These data show the permanence of mean state changes in climate on the region’s hydrology, with clear implications for the study site (an important water resource for ~500,000 people) and other lakes in the rapidly warming Andes.

## Introduction

**Figure 1:**
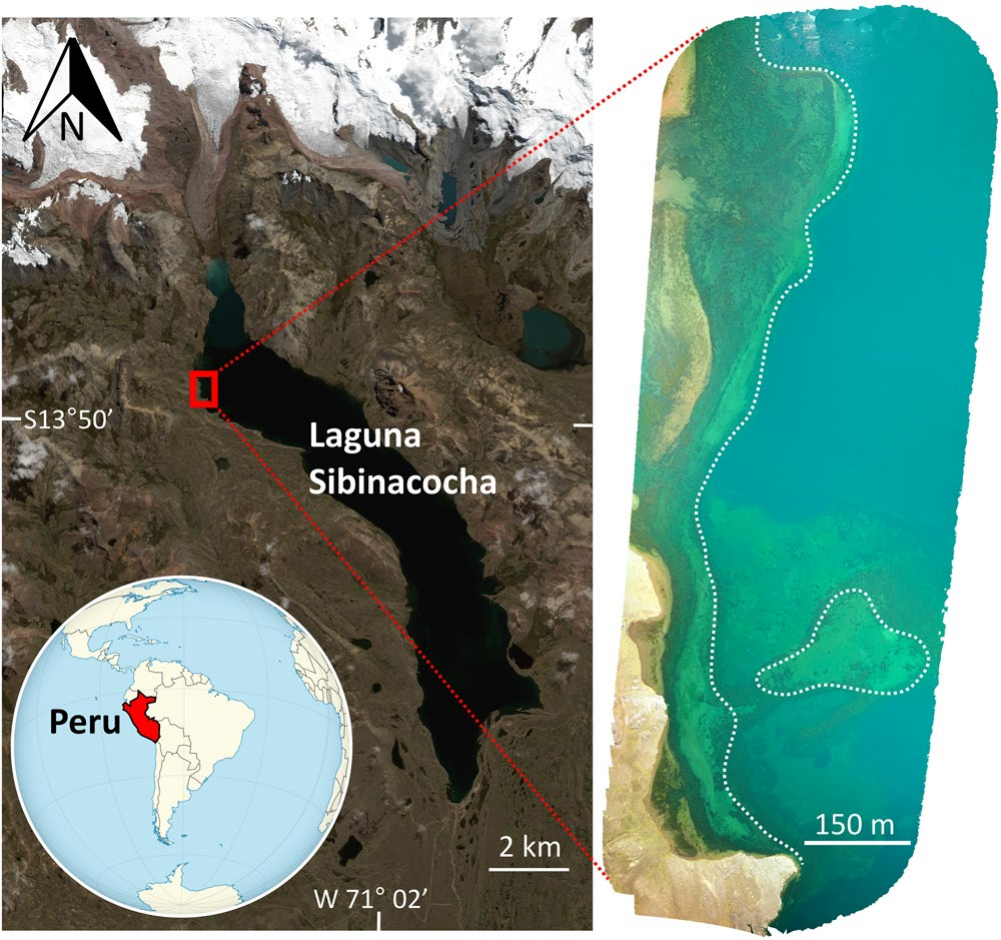
Location of Laguna Sibinacocha in the Peruvian Andes showing the region of the lake where the study pot was recovered (red rectangle). Unmanned aerial vehicle (UAV) imagery of the study region (right image) reveals a probable former shoreline prior to flooding, demarcated by a dotted white line. The paleo-shoreline generally follows the submerged beach berm running parallel to the current shoreline. Former channels and delta features are also evident in the upper portion of the image. The exact location of the study pot is not shown in order to protect the archaeological site. Satellite imagery of Laguna Sibinacocha and its surroundings was provided by DigitalGlobe Foundation. UAV imagery was processed using Maps Made Easy (https://www.mapsmadeeasy.com/).

Peru’s Cordillera Vilcanota mountain range is one of the world’s important water towers, supplying water to hundreds of thousands of people for agriculture, hydroelectricity, and household purposes. Laguna Sibinacocha stands out as a dominant feature of this landscape, even amongst the several mountain peaks that extend above 6,000 m asl and the massive Quelccaya Ice Cap. Situated at an altitude of 4,870 m asl, with an area of ~30 km^2^ and water depths over 90 m in places, there are few lakes of comparable size and elevation on the planet (Fig. 1). Laguna Sibinacocha is an important tributary to the Amazon River and a critical water supply to downstream communities and cities. Despite its cultural, ecological and societal significance, only recently has basic limnological data been provided on its water chemistry, maximum depth, and thermal regime^1^. Adding to the mystery of the lake is the recent discovery of pre-Hispanic artifacts and architecture submerged in its nearshore waters.

In 2011, archaeological remains were discovered under several meters of water in Laguna Sibinacocha. A dominant feature of this underwater site is a ~100 m long wall composed of rocks arranged in a zigzag (snake-like) pattern thought to indicate sacred architecture^2–4^. Its presence adjacent to Laguna Sibinacocha is compatible with the many depictions of serpents common to the pre-Hispanic iconography of the Lake Titicaca region, interpreted as being symbols of water-related deities^5,6^. Most of the structure appears to be constructed of a yellow stone (possibly dolomite) not present in the geology immediately surrounding the lake, which suggests the material may have been transported and further adds to the significance of this location as a potentially sacred site. Other artifacts submerged near the serpentine wall structure include intact pots, numerous pottery sherds and arrowheads. Similar artifacts, as well as mortuary monuments, have also been found on the surrounding landscape and tentatively date to multiple periods including the Formative Period in the Cuzco region (2500 BCE–200 CE), the Late Intermediate Period (~1000–1400 CE), the Inca Empire (~1400–1532 CE) and Colonial (1532–1800s CE). As archaeological investigations proceed, it seems apparent that this site held cultural significance to pre-Hispanic populations.

**Figure 2:**
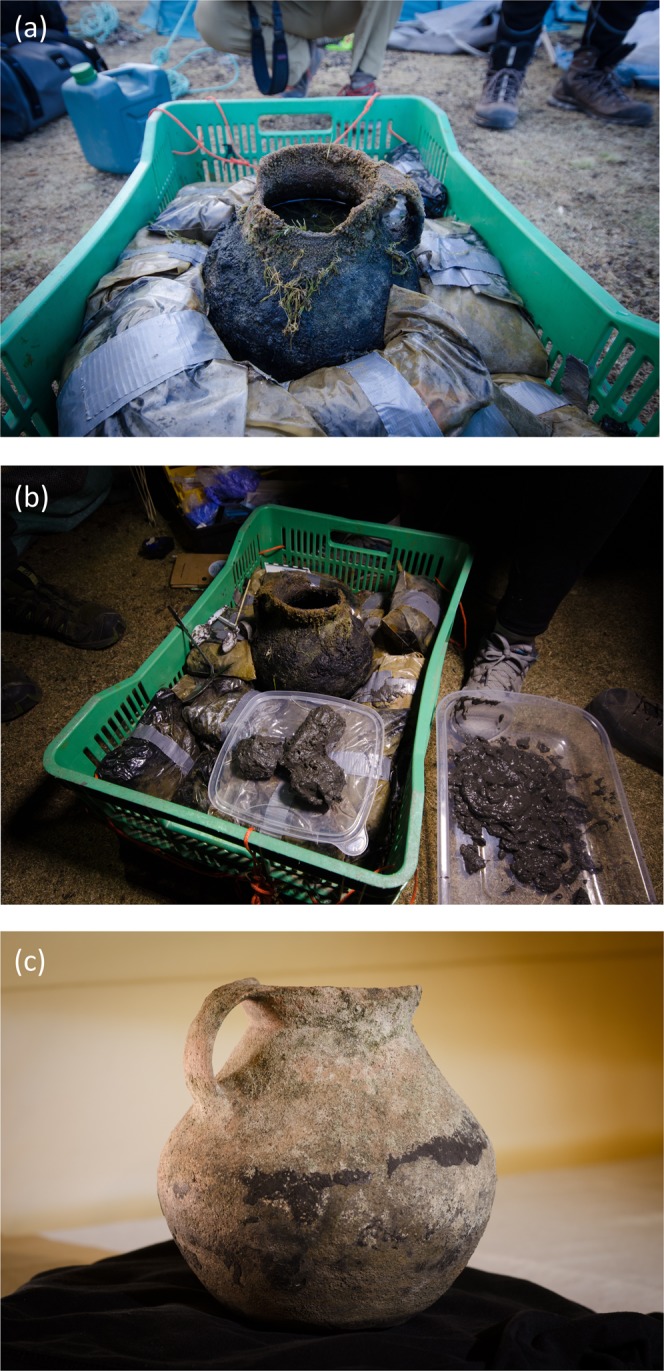
Images of the pre-Inca study pot and its contents. Images show the pot (**a**) immediately after its extraction from Laguna Sibinacocha; (**b)** with the three rocks recovered from its bottom in their original positioning; and (**c**) following cleaning and conservation.

As part of ongoing efforts to characterize the submerged ruins, an intact pot was recovered from adjacent to the zig-zag rock structure (Fig. 2). Besides accumulated sediments, the pot contained one elongated and two semi-round stones, which were arranged in a pattern suggestive of a phallus (Fig. 2b). Based on its stylistic typology and method of construction, the pot was identified as being most likely from the Late Intermediate Period (~1000–1400 CE), but could also be from a local ceramic tradition contemporaneous with the Inca Empire. Importantly, the upright pot had steadily accumulated lake sediment over time and thus preserved a natural archive of past change since submergence. The shallow water location of the pot is more sensitive to lake-level fluctuations than a deep water site. Thus, this unique sediment record has the potential to answer unknown questions regarding the history of this site and past hydrological variability that might not be attainable using the traditional approach of analyzing profundal zone sediments^7^, especially in a lake as large and deep as Laguna Sibinacocha.

Here, we use dated sediment intervals recovered from the extracted pot to address three questions. *First, when did rising water levels inundate the archaeological site at Laguna Sibinacocha?* We use the age of the basal material to demarcate the onset of sediment accumulation within the pot and, by extension, the timing of the lake-level rise. From an archaeological perspective, the basal age reveals whether the site was inundated during the Late Intermediate Period, or later. *Second, how have water levels fluctuated over time at Laguna Sibinacocha?* We use fossil diatom assemblages preserved within the pot sediments to inform about the nature of past hydrologic change. Diatoms (Bacillariophyceae) are algal bioindicators that respond sensitively to changes in lake-levels via alterations to habitat availability, light penetration, water chemistry, stratification and mixing regimes^8^. Thus, the composition of diatom assemblages will reveal, for example, whether water levels stabilized after the initial flooding, or if they fluctuated between high and low stands. *Third, what are the main environmental drivers responsible for the lake level rise at Laguna Sibinacocha?* We use high-resolution ice core records from the nearby Quelccaya Ice Cap^9^ to place paleolimnological data obtained from the pot sediments within a paleoclimatic context. This approach reveals the effects of mean state changes in climatic variables on lake water fluctuations at the study site, with clear management implications for this important water resource.

The pot submerged in Laguna Sibinacocha acted as an ersatz sediment trap preserving a history of limnological change since its inundation. This rare sedimentary archive provides long-term data on the past hydrological variability of an important water resource, all within the context of a newly discovered archaeological site in the Peruvian Andes.

## Results

### Radioisotope geochronology

**Figure 3:**
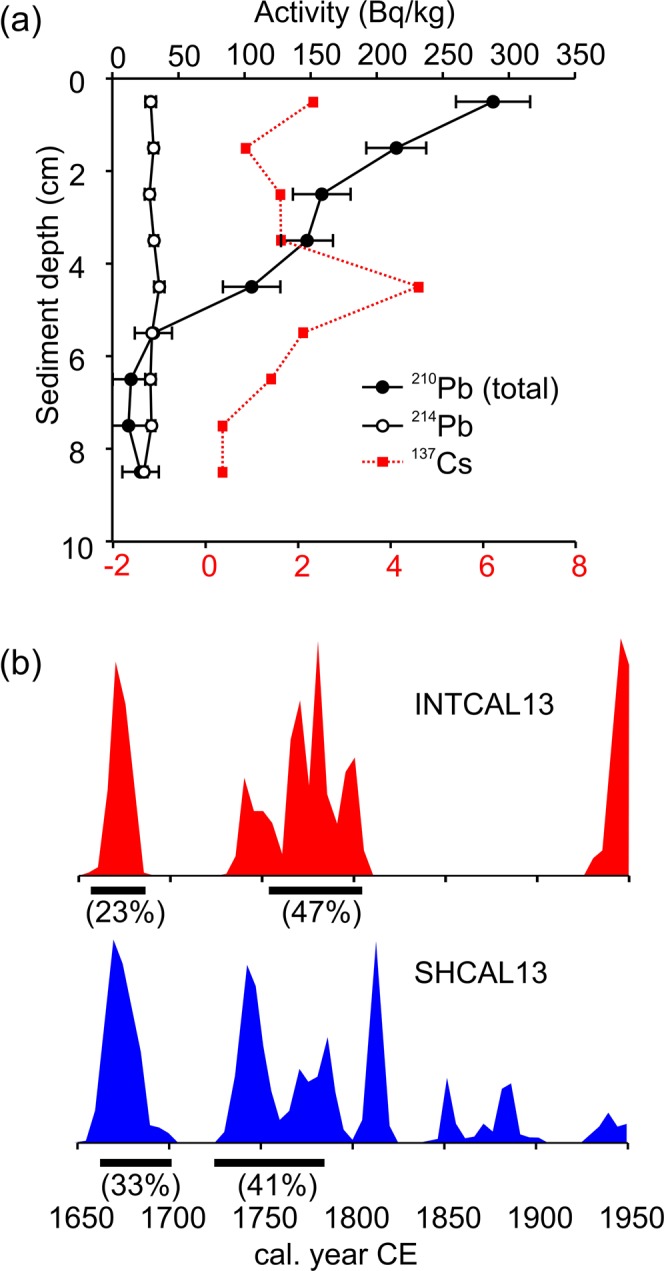
Plots showing radioisotopic dating results for the study pot sediments. (**a**) Total and supported ^210^Pb (as ^214^Pb) and ^137^Cs activity for the upper sediments. (**b**) Comparison of IntCal13 and SHCal13 calibrations for the herbaceous stem fragment recovered from the basal sediment. The black bar beneath each curve represents the relative area under the (2σ) probability distribution.

Total ^210^Pb activity shows a monotonic decline with depth until reaching supported levels at 5–6 cm depth (Fig. 3a). The steady decline in ^210^Pb activity indicates that lake sediment gradually accumulated in the pot over time, with minimal mixing. The ^137^Cs profile shows a slight, yet discernable, peak at the 4–5 cm depth interval (Fig. 3a). The 1963 CE dating horizon demarcated by this peak falls within the range of ^210^Pb dates that bracket this interval. We note that the maximum ^137^Cs activity recorded here is an order of magnitude lower than commonly reported in Andean lake sediments^10,11^. Thus, whether this peak represents the 1963 period of maximum fallout from weapons testing is debatable; however, it does seem to further confirm our ^210^Pb dating profile.

The herbaceous stem fragment isolated from the basal sediments gave a radiocarbon age of 190 ± 15 yr BP, which has multiple intersections with radiocarbon calibration curves from both the southern and northern hemispheres. The result is at least two probable separate calendar year age ranges from each calibration curve; however, both calibration curves produced age ranges that overlap (Fig. 3b, Table 1). The IntCal13 calibrated age ranges (2σ) with the highest relative areas under the probability distribution are 1663–1682 and 1761–1805 cal yr CE. The SHCal13 calibrated age ranges (2σ) with the highest relative areas under the probability distribution are 1668–1705 and 1721–1788 cal yr CE (Table 1). Although there is overlap, the IntCal13 curve generated possible age ranges that were both slightly younger and older than the SCHCal13 curve (Fig. 3b). If we accept the extremes of the oldest and youngest possible age ranges derived from IntCal13 (Table 1), then the 9-cm long sediment profile in the pot represents a possible range from ~280–420 years of limnological history.

**Table 1:** Radiocarbon dates showing comparison of IntCal13 and SHCal13 calibrations for the herbaceous stem fragment recovered from the basal pot sediments.

Radiocarbon age 190 ± 15 (UCIAMS # 199439)		
Calibrated age ranges (2σ)	Rel. area under	Age range (CE)
cal yr CE	prob. distr.	adj. for 2017
**IntCal13**		
1663–1682	0.233	1596–1615
1737–1757	0.113	1670–1690
1761–1804	0.470	1694–1737
1936–1949*	0.185	
**SHCal13**		
1668–1705	0.327	1601–1638
1721–1785	0.411	1654–1718
1793–1811	0.148	1726–1744
1837–1846	0.026	
1859–1863	0.005	
1866–1879	0.045	
1929–1949*	0.039	

*Considered suspect due to impingement on the end of the calibration data set.

### Sub-fossil diatom assemblages

**Figure 4:**
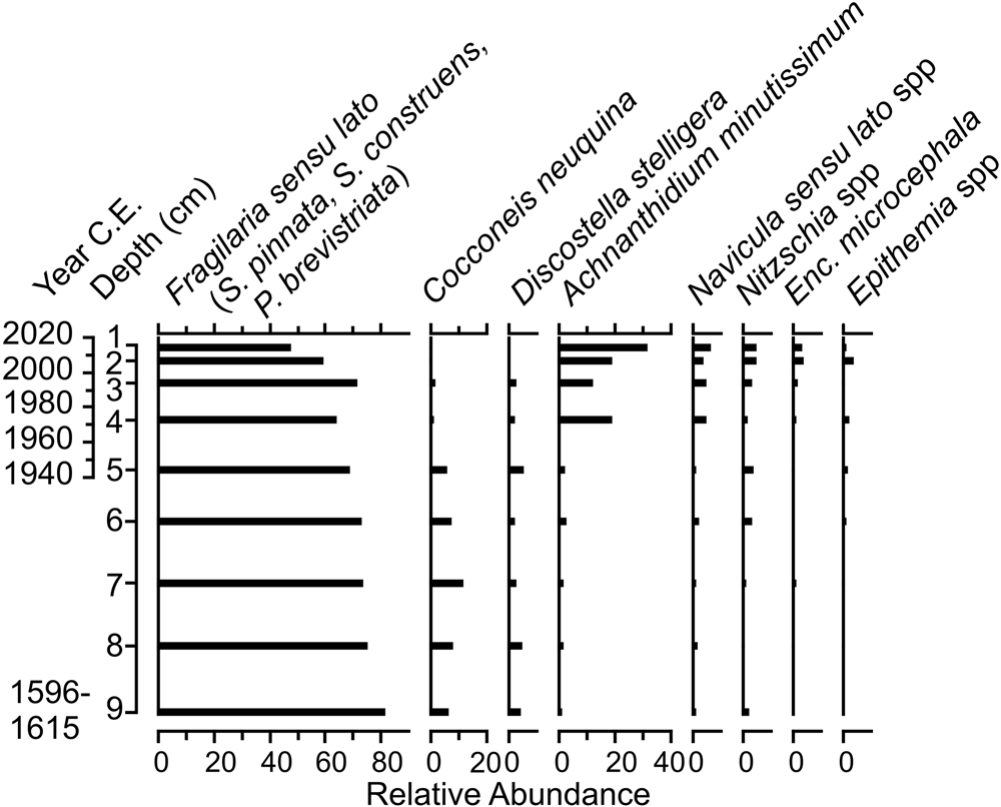
Sub-fossil diatom assemblage profile from the pot sediments recovered from Laguna Sibinacocha. The age profile on the left-hand side is based on the constant-rate-of-supply (CRS) model using excess ^210^Pb activities for the upper ~5 cm, and the basal ^14^C date at 9-cm depth is the oldest probable age range from IntCal13. The genus *Encyonema* is abbreviated as “Enc”.

The oldest sediments were dominated (~80% relative abundance) by small benthic fragilarioids primarily composed of *Staurosirella pinnata* Ehrenberg, *Staurosira construens* Ehrenberg, and *Pseudostaurosira brevistriata* Grunow. *Cocconeis neuquina* Frenguelli is the only non-fragilarioid taxon to exceed 5% relative abundance in the bottom portion of the record. At 3.5 cm depth (~1972 CE), and continuing to the surface sediments, diatom diversity increases driven by higher relative abundances of *Achnanthidium minutissimum* (Kützing) Czarnecki, and to a lesser extent *Navicula sensu lato* spp, *Nitzschia* spp, *Epithemia* spp, and *Encyonopsis microcephala* (Grunow) Krammer. *Discostella stelligera* (Cleve & Grunow) Houk & Klee is the only planktonic taxon documented in appreciable quantities (~5% relative abundance) throughout the record (Fig. 4).

## Discussion

The age of the basal sediment in the pot provides the best estimate of when rising water levels flooded the archaeological remains in Laguna Sibinacocha. This, of course, presupposes that the study pot was *in situ* during flooding and not thrown or lowered into the lake at a later date. In early Andean cultures, it was commonplace to cast offerings such as pottery, animal bones, and figurines into lakes^12^. Offerings to lakes were made to ensure the fertility of llama and alpaca herds, as it was widely believed these animals originated from lakes^13^. In Lake Titicaca, stone containers containing gold and silver figurines of llamas, female statues, tupus (shawl pins) and other artifacts of Inca origin were found submerged near ceremonial sites and are believed to have been lowered into place after submergence of the site^12^.

In Laguna Sibinacocha, the onset of sediment accumulation in the study pot is difficult to pinpoint because the ^14^C age intercepts the IntCal13 and SHCal13 calibration curves multiple times resulting in at least two probable calendar year age ranges for each curve (Table 1, Fig. 3b). However, even if we accept the oldest age range of 1596–1615 CE (adjusted for time zero at 2017 CE), this indicates sediment began accumulating ~60–80 years after the end of Inca reign, negating the possibility that Incas cast the pot into the lake after the flood. The question arises whether local Quechua people, who likely carried out the traditions of the Inca well after their demise^14,15^, could have placed the pot into the lake as an offering after submergence of the site. This seems improbable given that the pot was recovered ~50 m from the modern shoreline making too far a distance to throw from the water’s edge. Also, with no evidence of boats or rafts in this region, it is unlikely that the Quechua would have waded or swam this far and deep into the lake to place an offering.

Andean cultural practices offer an alternate hypothesis that might account for an offset between the pot’s basal sediment date and the timing of lake flooding. Based on the pot’s proximity to what appears to be sacred architecture, and the stones found inside it, a logical interpretation is that it was left as an offering. Precious objects were often wrapped in cloth prior to being placed as offerings^16^. Therefore, it is possible that the pot was covered with some sort of textile when it was placed on the historical shoreline prior to the site’s immersion. In this scenario, sediment accumulation would have been delayed until the textile had decomposed. However, no remnants of fabrics were found within the pot, which may be expected if it was completely wrapped in cloth.

Although there are cultural explanations that could account for a delay in the timing of when the pot began to accumulate sediment relative to the onset of rising water levels, Occam’s razor indicates the pot was *in situ* and uncovered during the lake-level rise. The multiple intersections of the basal ^14^C age with the radiocarbon calibration curves present a range of possible dates for the inundation of the site spanning from the early- 1600s to the mid-1700s CE (Table 1, Fig. 3b).

The radioisotopic data confirm that the sediment record within the pot is stratigraphically intact (Fig. 3a,b). The absence of any reversals or plateaus in the ^210^Pb profile suggests a stable depositional environment and indicates the pot was submerged deep enough to avoid disturbance by wave action. The presence of *Discostella stelligera* throughout the entire sediment record, albeit at low (~5%) relative abundances, confirms that overlying water was of sufficient depth to support populations of planktonic diatoms (Fig. 4). The complacent fossil diatom profile, excepting the post-1970s changes (discussed below), reflect stable limnological conditions. If water levels in Laguna Sibinacocha frequently altered between high and low stands, to the point of exposing the pot to the surface, we would expect to see shifts in the fossil assemblages, such as the presence of aerophilic taxa and, certainly, the absence of obligate planktonic species. Also, if the pot was located above or near the surface of the water, wave action would have undoubtedly resulted in a mixed ^210^Pb profile. Thus, the radioisotopes and fossil diatom assemblages both indicate that the inundation of the archaeological site occurred relatively rapidly and that water levels have remained consistently high until present-day.

The sub-fossil diatom assemblages from the study pot reflect the limnological conditions of Laguna Sibinacocha. The *Fragilaria sensu lato* complex that dominate the diatom profile (Fig. 4) are consistent with their high abundances in cold, circumneutral-to-alkaline lakes in Arctic and alpine regions^1,17–20^. *Cocconeis neuquina*, the only non-fragilarioid taxon to exceed 5% relative abundance prior to ~1970 CE, has been documented as a periphytic taxon in shallow Patagonian lakes characterized by circumneutral to alkaline pH, with generally cool temperatures (1.7 to 16.4 °C) and varying conductivity (37–1390 µS/cm)^21^.

The increase in *A. minutissimum* and, to a lesser extent, *Encyonopsis microcephala* and species of *Navicula*, *Nitzschia*, and *Epithemia* in the post-1970s sediments likely reflect a habitat change occurring within the pot itself, rather than a lake-wide change in aquatic conditions. A sediment core from the southern basin of Laguna Sibinacocha, recovered in 30 m depth and spanning the last ~85 years, records a complacent diatom profile dominated by the same *Fragilaria sensu lato* complex identified in the pot, but no post-1970s increase in diversity. This confirms the absence of any lake-wide ecological change during this period (Supplementary Fig. S1). At present, the macroalga *Chara* dominates the lake bottom from where the study pot was recovered. We hypothesize that the increased diatom diversity in the surface sediments reflects the establishment of macrophyte beds in the immediate region. Similar assemblage shifts have been observed in sediment records from Arctic regions where climate-induced changes to aquatic habitat (e.g., increased mosses) resulted in a shift from small benthic fragilarioids to higher diversity assemblages including epiphytic as well as stalked and tube-dwelling species^22,23^. Although the mechanism responsible for enhanced macrophyte growth in Arctic lakes (i.e., reduced ice cover and longer growing season) is not a factor in the perennially ice-free Laguna Sibinacocha, the assemblage shift is consistent with a change in habitat from a largely sediment-based substrate to one dominated by macrophytes.

**Figure 5:**
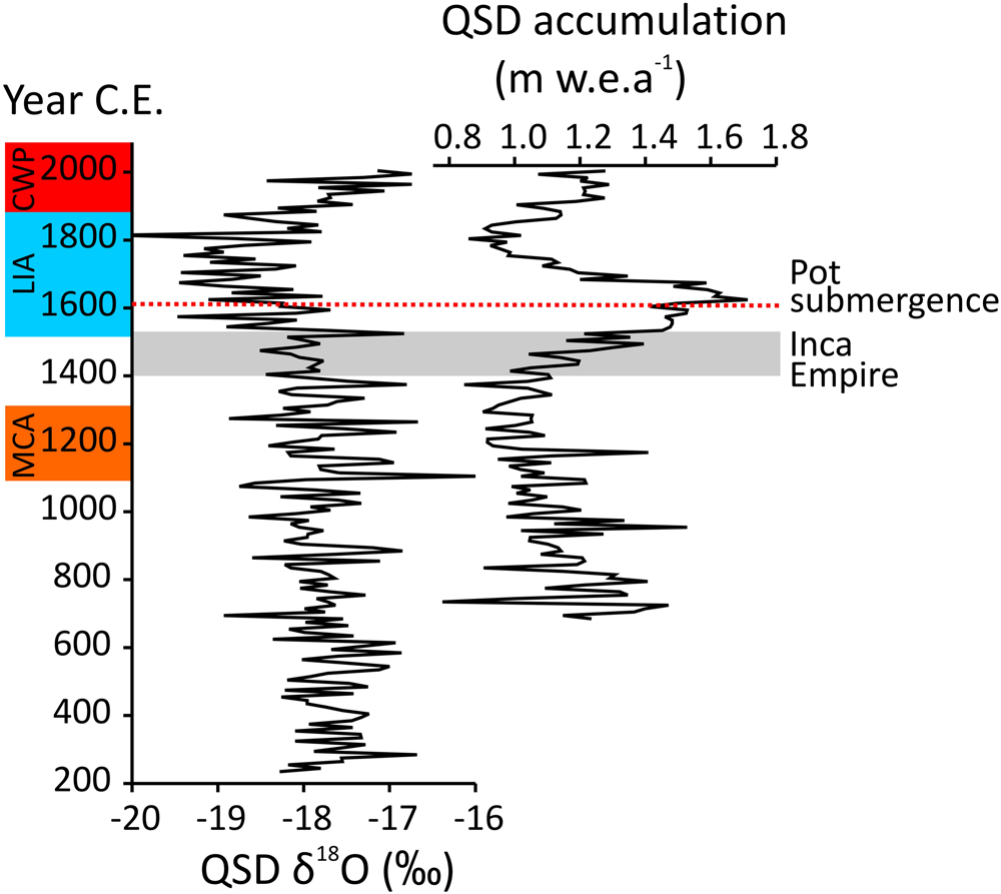
Decadal averages of δ^18^O and net accumulation from the Quelccaya Summit Dome (QSD) ice core^9^ showing specific climatic periods including the Medieval Climate Anomaly (MCA), Little Ice Age (LIA), and Current Warm Period (CWP) on the left margin. The time period encompassed by the Inca Empire (1400 – 1532 CE) is shown by the grey bar. The dashed red line indicates the onset of the inferred submergence of the study pot.

Annually-resolved ice core records from the nearby Quelccaya Ice Cap (QIC) provide paleoclimatic context with which to interpret lake level changes in Laguna Sibinacocha^9^. Variability in precipitation over centennial to millennial timescales certainly has the potential to alter lake levels in the Andes^24,25^. An extreme example occurred in Lake Titicaca during a prolonged drought period between 6,000 to 5,000 years BP that caused water levels to drop by 85 m below present-day levels^26^. In the Quelccaya record, δ^18^O values, which are thought to reflect variability in air temperatures over long-term timescales^27^, show a well-defined Little Ice Age (LIA) at ~1520–1880 CE (Fig. 5). The annual net accumulation (Fig. 5), which represents a regional signal of precipitation variability, records high values in the early LIA (1520–1680 CE) and low values in the late LIA (1681–1880 CE)^9^.

Both ^14^C calibration curves generated age ranges that overlap, but because the curves produced multiple intercepts there exists the probability of at least two likely age ranges (Table 1, Fig. 3b). Therefore, the choice of the most appropriate curve is less critical than determining the most probable age of the basal sediment. Both curves indicate a high probability that the pot became inundated, and started to accumulate sediment, in the early- to mid-1700s CE (Table 1, Fig. 3b). However, in this region, the early- to mid-1700s CE was a period of cool temperatures and below average precipitation (Fig. 5); hardly conditions that that would be conducive to a large lake level rise. If we accept the older estimate of the two most probable age ranges as the timing of pot submergence (i.e., early- 1600s CE), then the inundation of the archaeological site would have followed nearly a century of elevated precipitation^9^, in the middle of the wettest period of the last ~1,300 years (Fig. 5). Given the paleoclimatic context, we favor the interpretation that the waters of Laguna Sibinacocha rose by several meters starting in the early-1600s CE.

The Quelccaya ice accumulation record shows persistently low precipitation levels beginning at 1180 CE and continuing into the Inca Empire (Fig. 5)^28^. Drier than average conditions would result in lower-than-present lake levels allowing pre-Hispanic populations to occupy the lower shorelines of Laguna Sibinacocha. Unmanned aerial vehicle (UAV) imagery of the study region shows the likely paleo-shoreline available to the pre-Hispanic populations prior to flooding (Fig. 1). The image reveals a shallow submerged area that shares a similar morphology to the modern shoreline. The former shoreline is approximately delineated by a beach berm that runs parallel to the current shoreline and the lack of any apparent modification of the submerged area is consistent with relatively recent flooding (i.e., past few centuries) as constrained by the ^14^C age of the pot’s basal sediment.

Just prior to the end of the Inca reign at 1532 CE, the region entered a ~160-year period of wetter than average conditions^9,29^, driven in part by a prolonged intensification of the South American summer monsoon rainfall^30,31^. The basal pot date indicates that the wet phase of the early LIA raised water levels in Laguna Sibinacocha and eventually inundated the study pot and surrounding archaeological features beginning in the early- 1600s CE. Although precipitation declined after 1680 CE, cool temperatures during the LIA would have mitigated evaporation from the lake’s surface and kept water levels elevated. This is supported by an undisturbed ^210^Pb profile and fossil diatom data indicating water levels remained high above the pot since its inundation.

Following 1880 CE, the QIC record shows a return to generally wetter conditions relative to the dry phase of the late-LIA^9^, concurrent with warming that is unprecedented for at least the last two millennia (Fig. 5)^27^. The effect of this warming over the last several decades on the Cordillera Vilcanota has resulted in an upward range expansions of soil microbes and pathogens^32^, new elevation records for amphibian species^33^, and massive reductions in the cryosphere^34^. Glaciers draining into Laguna Sibinacocha have been in recession since at least 1931 CE as documented by aerial photography and satellite imagery, with some ice margins now receding at an average rate of 13 m/year over the past decade^33^. This increase in glacial meltwater has kept water levels high and the archaeological site in this study hidden from view since lake levels rose ~400 years ago.

In Laguna Sibinacocha, a rare sedimentary archive from a pre-Inca pot reveals linkages between past changes in climate, hydrology, and Andean culture. Knowledge that lake level fluctuations have concealed pre-Hispanic ruins as large as a 100 m long serpentine rock structure for the past four centuries teases at what other archaeological remains the lake may contain. The timing of the lake-level rise during a wet phase of the LIA provides evidence that the lake and its watershed are susceptible to large changes in hydrology. The persistence of high water levels in Laguna Sibinacocha for the past four hundred years demonstrates the permanence that a mean state change in hydrology can have on lake water levels in this region. This has implications to the present-day, especially given the climate-related changes occurring within the Cordillera Vilcanota and the role of Laguna Sibinacocha as a critical water resource to hundreds of thousands of people in downstream communities.

## Materials and Methods

### Site description

Laguna Sibinacocha (13°49′26.44″S; 71°04′26.44″W) is a large (~30 km^2^) and deep (>90 m) lake located at an altitude of 4,870 m asl in the Cordillera Vilcanota range of southeastern Peru (Fig. 1). The lake is a primary source of the Vilcanota-Urubamba River, a major tributary to the Amazon River. Laguna Sibinacocha is circumneutral (pH = 7.9), ultra-oligotrophic (total phosphorus = 3.3 µg/L) and relatively dilute (conductivity = 380 µS/cm). It is a cold-water lake (temperatures < 12 °C) that is well-mixed with only brief periods of weak thermal stratification^1^. An actively melting glacier^33^ drains into the northern basin of the lake but this does not result in any temperature gradient along its ~15 km length^1^.

In 1996, a dam was constructed at the outflow of Laguna Sibinacocha in order to ensure adequate water supply for downstream populations. Water supplied from Laguna Sibinacocha is primarily used for agriculture (50%), followed by energy (36.5%) and households (11.6%)^35^. Aerial photographs of the lake taken in 1931^36^ show that the underwater ruins, including the serpentine rock structure, were submerged prior to the construction of the dam. Hydrological information on Laguna Sibinacocha is difficult to obtain; however, we observed high water marks of ~2 m during the end of the dry season when lake levels record their annual minimums. During the low water stand in the 2017 field season, the archaeological site was submerged at ~3 m depth.

In the Cordillera Vilcanota, annual temperature variability is minimal with only 1–2 °C separating the wet austral summer (October to March) from the dry winter (April to September)^37^. However, diurnal temperatures can vary by as much as 18 °C. Precipitation shows strong seasonality with over 70% occurring from December to March. The nearby Ccatcca meteorological station (3,729 m asl), ~60 km west-northwest of Laguna Sibinacocha, records mean minimum and maximum daily temperatures of 1.3 and 15.3 °C, respectively, and a mean annual precipitation of 608 mm (1965–2014). The Pomacanchi meteorological station (3,200 m asl), ~60 km west-southwest of Laguna Sibinacocha, records mean minimum and maximum daily temperatures of 2.8 and 17.2 °C, respectively, and mean annual precipitation of 851 mm (1985–2014). The Ccatcca and Pomacanchi climate stations record significant (P < 0.01) warming trends for both maximum and minimum daily temperatures over their period of measurement^1^.

### Sample recovery and processing

A dive team working with an underwater archaeologist and artifact conservation specialist recovered an intact pre-Hispanic pot from ~3 m water depth and ~50 m from shore in the northern portion of Laguna Sibinacocha (Fig. 2). The pot contained sediments extending to a depth of 9 cm. The bottom of the pot contained three large stones arranged in the shape of a phallus (Fig. 2). The surface sediment had abundant growth of the macroalga *Chara*, which dominates the littoral zone from where the pot was recovered. Using a modified spoon, sediment was removed at 1-cm intervals and placed into Whirlpack bags.

Geochronology on the upper portion of the profile was established with a constant-rate-of-supply (CRS) model using excess ^210^Pb activities, and verified using the anthropogenic isotope ^137^Cs. Sediment was counted on a digital high-purity germanium spectrometer (DSPec, Ortec) with a well-type gamma detector at Queen’s University consisting of a germanium crystal with lithium diffused electrodes. The CRS dates were developed using the ScienTissiME package in MatLab^38^.

A basal sediment age, demarcating the onset of sediment accumulation, was obtained on an herbaceous stem macrofossil isolated by A. Telka of Paleotec Services and dated by Accelerator Mass Spectrometry (AMS) radiocarbon (^14^C) at the Keck Carbon Cycle Accelerator Mass Spectrometry Laboratory at the University of California, Irvine Earth System Science Department. The radiocarbon age was calibrated to years before present (cal yr BP, where BP = 1950) using the program Calib Rev 7.0.4^39^. In order to better align with the ^210^Pb chronology, the calibrated date was adjusted for zero age at 2017 CE. Age ranges are presented using calibration curves from both the Northern (IntCal13)^40^ and Southern (SHCal13)^41^ hemispheres. Choosing an appropriate calibration curve for ^14^C dates is complicated in the tropical Andes because this region receives air masses from both hemispheres, which have different concentration of ^14^C. This is further complicated by the fact that the division between hemispheric air masses is the Intertropical Convergence Zone, which is not fixed but rather moves seasonally as well as over long-term timescales^42^. Mixed calibration curves offer a promising solution^43^, although there is no way to gauge the relative contributions from each hemisphere. The approach we take here is to present age ranges from both northern and southern hemispheres, which avoids any bias in selecting ages that best fit our data or preconceptions^44^.

Preparation of sediment for diatom microfossil analysis followed standard techniques^45^. Diatoms were examined using a 100x oil immersion objective (numerical aperture = 1.3) and a 10x ocular, with condenser lens on a Leica DMRB microscope equipped with differential interference contrast (DIC) optics. For each interval of the 9-cm record, a minimum of 300 valves was identified to species level, or variety, where possible.

### Aerial imaging

A high resolution, georeferenced aerial image of the submerged study area was obtained using a DJI Mavic 2 Pro unmanned aerial vehicle (UAV) equipped with a Hasselblad L1D-20c camera. The flight path was programmed using Map Pilot and flown at an altitude of 185 m. A composite image was then generated from 186 Nadir images that were captured from over the 78.3 hectare area of interest and processed using Maps Made Easy.

## Supplementary information

Supplementary Information
