# Author Correction: A pre-Inca pot from underwater ruins discovered in an Andean lake provides a sedimentary record of marked hydrological change

**DOI:** 10.1038/s41598-020-66186-4

**Published:** 2020-06-16

**Authors:** Neal Michelutti, Preston Sowell, Pedro M. Tapia, Christopher Grooms, Martin Polo, Alexandra Gambetta, Carlos Ausejo, John P. Smol

**Affiliations:** 1 Paleoecological Environmental Assessment and Research Laboratory (PEARL), Department of Biology, Queen’s University, Kingston, Ontario Canada; 2 Geotic Solutions, Boulder, Colorado, USA; 3 INAIGEM – Dirección de Investigación en Ecosistemas de Montañas, Huaraz, Perú; 4 El Centro Peruano de Arqueología Marítima y Subaquática [CPAMS], Lima, Peru; 5 Kullasiri S.A.C., Lima, Peru

Correction to: *Scientific Reports* 10.1038/s41598-019-55422-1, published online 16 December 2019

The original version of this Article contained an error in Table 1 where there was no age adjustment necessary to the original radiocarbon ages presented in the first column. This has now been corrected. Where it was previously inferred that a lake level rise occurred in the early 1600s, now with the correct age this changes to the late 1600s CE. This change does not change the interpretation of the data.

However, as a result the dates of pot submergence referred to in the text have been revised. In the Abstract,

“The pot’s basal sediment age places the timing of lake-level rise at ~1600 CE, which post-dates the end of the Inca Empire (1400–1532 CE) by several decades.”

now reads:

“The pot’s basal sediment age places the timing of lake-level rise during the late ~1600s CE, which post-dates the end of the Inca Empire (1400–1532 CE) by approximately 150 years.”

In the Results section, under the subheading ‘Radioisotope geochronology’,

“If we accept the extremes of the oldest and youngest possible age ranges derived from IntCal13 (Table 1), then the 9-cm long sediment profile in the pot represents a possible range from ~280–420 years of limnological history.”

now reads:

“If we accept the extremes of the oldest and youngest possible age ranges derived from IntCal13 (Table 1), then the 9-cm long sediment profile in the pot represents a possible range from ~215–350 years of limnological history.”

In the Discussion,

“However, even if we accept the oldest age range of 1596–1615 CE (adjusted for time zero at 2017 CE), this indicates sediment began accumulating ~60–80 years after the end of Inca reign, negating the possibility that Incas cast the pot into the lake after the flood.”

now reads:

“However, even if we accept the oldest age range of 1663–1682 CE, this indicates sediment began accumulating ~130–150 years after the end of Inca reign, negating the possibility that Incas cast the pot into the lake after the flood.”

“The multiple intersections of the basal ^14^C age with the radiocarbon calibration curves present a range of possible dates for the inundation of the site spanning from the early- 1600s to the mid-1700s CE (Table 1, Fig. 3b).”

now reads:

“The multiple intersections of the basal ^14^C age with the radiocarbon calibration curves present a range of possible dates for the inundation of the site spanning from the late- 1600s to the early-1800s CE (Table 1, Fig. 3b).”

“Both curves indicate a high probability that the pot became inundated, and started to accumulate sediment, in the early- to mid-1700s CE (Table 1, Fig. 3b).”

now reads:

“Both curves indicate a high probability that the pot became inundated, and started to accumulate sediment, in the mid- to late-1700s CE (Table 1, Fig. 3b).”

“If we accept the older estimate of the two most probable age ranges as the timing of pot submergence (i.e., early- 1600s CE), then the inundation of the archaeological site would have followed nearly a century of elevated precipitation^9^, in the middle of the wettest period of the last ~1,300 years (Fig. 5). Given the paleoclimatic context, we favor the interpretation that the waters of Laguna Sibinacocha rose by several meters starting in the early-1600s CE.”

now reads:

“If we accept the older estimate of the two most probable age ranges as the timing of pot submergence (i.e., late- 1600s CE), then the inundation of the archaeological site would have followed approximately a century and a half of elevated precipitation^9^, in the middle of the wettest period of the last ~1,300 years (Fig. 5). Given the paleoclimatic context, we favor the interpretation that the waters of Laguna Sibinacocha rose by several meters starting in the late-1600s CE.”

“The basal pot date indicates that the wet phase of the early LIA raised water levels in Laguna Sibinacocha and eventually inundated the study pot and surrounding archaeological features beginning in the early- 1600s CE.”

now reads:

“The basal pot date indicates that the wet phase of the early LIA raised water levels in Laguna Sibinacocha and eventually inundated the study pot and surrounding archaeological features beginning in the late-1600s CE.”

“This increase in glacial meltwater has kept water levels high and the archaeological site in this study hidden from view since lake levels rose ~400 years ago.”

now reads:

“This increase in glacial meltwater has kept water levels high and the archaeological site in this study hidden from view since lake levels rose ~350 years ago.”

In the Materials and Methods, under the subheading ‘Sample recovery and processing’, the statement “In order to better align with the ^210^Pb chronology, the calibrated date was adjusted for zero age at 2017 CE” has been removed, therefore,

“The radiocarbon age was calibrated to years before present (cal yr BP, where BP  =  1950) using the program Calib Rev 7.0.4^39^. In order to better align with the ^210^Pb chronology, the calibrated date was adjusted for zero age at 2017 CE. Age ranges are presented using calibration curves from both the Northern (IntCal13)^40^ and Southern (SHCal13)^41^ hemispheres.”

now reads:

“The radiocarbon age was calibrated to years before present (cal yr BP, where BP  =  1950) using the program Calib Rev 7.0.4^39^. Age ranges are presented using calibration curves from both the Northern (IntCal13)^40^ and Southern (SHCal13)^41^ hemispheres.”

