# Supplementary figures and images for: A pre-Inca pot from underwater ruins discovered in an Andean lake provides a sedimentary record of marked hydrological change
Source: Sci Rep. 2019 Dec 16;9:19193. doi: 10.1038/s41598-019-55422-1 (PMC6915777; doi:10.1038/s41598-019-55422-1)

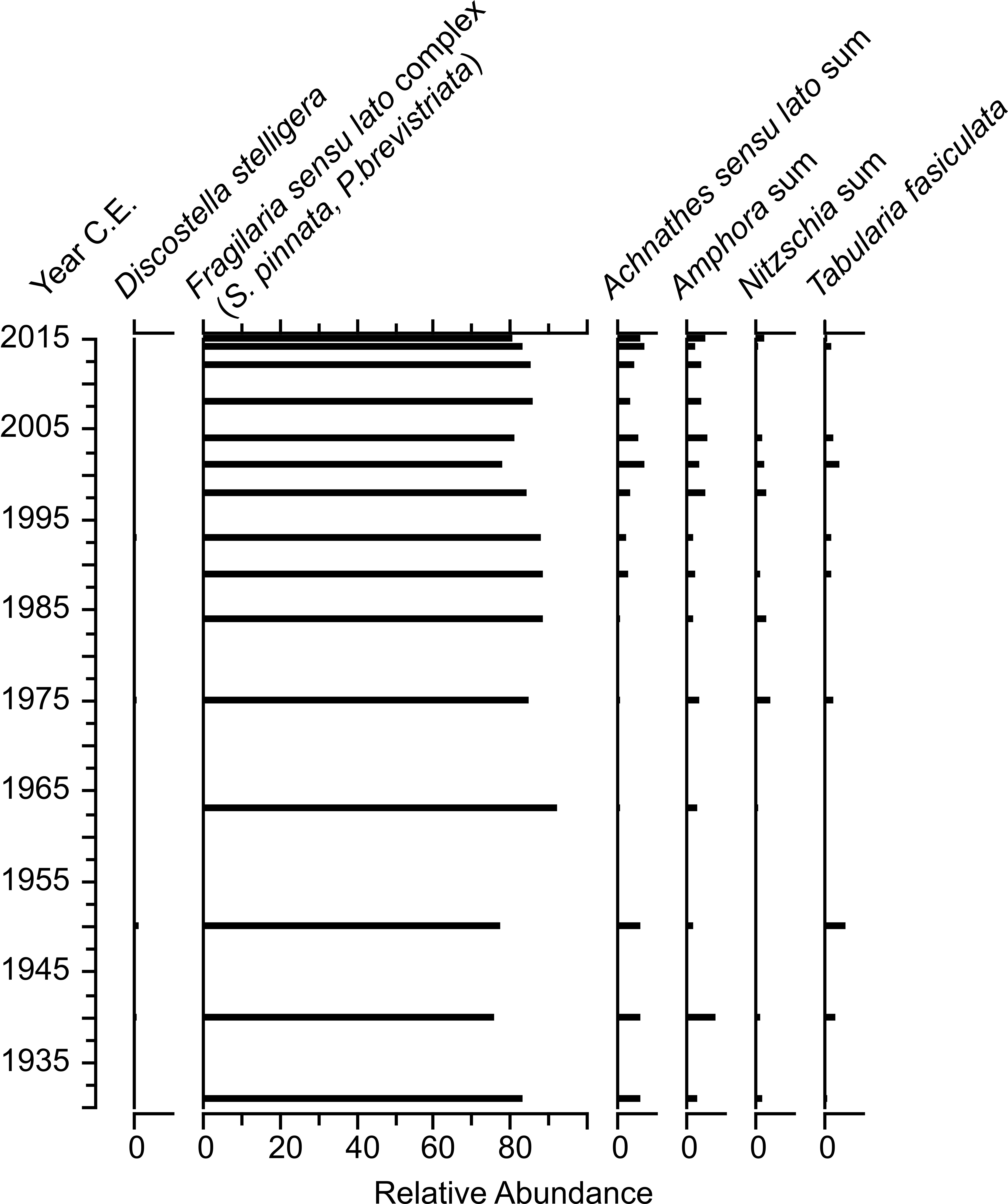

Supplement: Supplementary file 1 — Supplementary Information [file 41598_2019_55422_MOESM1_ESM.jpg]
